# Enhanced urethral identification for radiotherapy planning using fat-suppressed 3D T2-weighted magnetic resonance imaging

**DOI:** 10.1007/s12194-025-00903-4

**Published:** 2025-04-01

**Authors:** Yutaka Kato, Takayoshi Nakaya, Kuniyasu Okudaira, Yumiko Noguchi, Mariko Kawamura, Shunichi Ishihara, Shinji Naganawa

**Affiliations:** 1 Department of Radiological Technology, Nagoya University Hospital, 65 Tsurumai-cho, Shouwa-ku, Nagoya, Aichi 466-8560 Japan; 2 Department of Radiology, Nagoya University Graduate School of Medicine, 65 Tsurumai-cho, Shouwa-ku, Nagoya, Aichi 466-8560 Japan

**Keywords:** Urethra, Prostate, Brachytherapy, Magnetic resonance imaging, Radiotherapy planning

## Abstract

This study proposes a fat-suppressed three-dimensional T2-weighted (3D-T2W) sequence on magnetic resonance imaging to enhance prostatic urethral identification in radiotherapy planning. Conventional 3D-T2W and the proposed sequence were obtained to evaluate prostatic urethral identification in 13 male patients. The proposed sequence demonstrated significantly higher Dice similarity coefficients compared to conventional 3D-T2W sequence (*p* = 0.001) and superior contrast-to-noise ratios. The proposed sequence also achieved significantly better visibility scores in visual assessment (*p* = 0.001). The proposed technique uses fat suppression in a standard 3D-T2W sequence, making it a simple and clinically applicable method that does not require specialized sequence designs. Our findings suggest that this approach could be a valuable noninvasive method for enhancing prostatic urethral identification, although further research with larger sample sizes and optimization of acquisition parameters is needed.

## Introduction

Accurate prostatic urethral identification is an important factor in reducing the risk of urinary toxicity in radiation therapies such as stereotactic body radiotherapy or brachytherapy [1–3]. The most reliable approach for urethral identification is the insertion of a Foley catheter [4, 5]; however, catheterization every time for multiple days of irradiation is invasive, associated with a risk of infection [6, 7], and is undesirable for patients. Furthermore, urethral catheter placement may displace the urethral position [8, 9] and catheter removal may cause prostate rotation [10], resulting in possible planning inaccuracies.

Several studies have attempted to utilize magnetic resonance imaging (MRI) without catheterization. Most of these studies investigated with two-dimensional (2D) T2-weighted (T2W) turbo spin-echo (TSE) sequences [11–14]. In recent years, several studies have been conducted using three-dimensional (3D) sequences to improve urethral identification performance [15–17]. Kato and Okumiya et al. demonstrated that the 3D sequence had superior urethral identification compared to 2D, but the accuracy was inferior to that of computed tomography (CT) with catheter insertion [17]. Therefore, no complete approach exists for noninvasive prostatic urethral identification, and developing sequences with improved urethral identification is desirable for accurate radiotherapy planning.

The prostatic urethra is normally visualized as a high-intensity tract on T2W images. However, standard T2W sequences may be hindered in urethral identification because of the high signal intensity of the surrounding fat. The fat suppression technique is commonly used in clinical MRI examinations [18, 19], the dynamic range of the image display can alter if the high signal intensity of fat is suppressed, contributing to an improved tissue contrast. Therefore, we hypothesized that a fat-suppressed 3D-T2W image would allow for superior urethral identification compared to standard 3D-T2W. However, to our knowledge, no study has evaluated urethral identification using fat-suppressed T2W images. This study aimed to propose a novel fat-suppressed 3D-T2W sequence for accurate prostatic urethral identification.

## Materials and methods

### Patient data

This study was approved by the Ethics Committee of our institution. The requirement for informed consent was waived by the committee, because the clinical MRI data were collected retrospectively. The total number of included subjects was 13 male patients (mean age, 69.5 years; range, 57–81 years) with prostate cancer who received radiotherapy between October 2022 and June 2023 at our hospital. All participants underwent an MRI without a Foley catheter.

### Image acquisition

All MRI scans were performed using a 3 T scanner (MAGNETOM Skyra, Siemens, Erlangen, Germany) with an 18-channel body and a spine matrix coil. Two sequences were obtained for the prostatic urethra identification. One was a standard 3D-T2W image with sampling perfection and application-optimized contrasts using a different flip-angle evolution (SPACE) sequence, which is our routine protocol for radiotherapy planning (conventional sequence). The second is a newly proposed sequence that is more specialized for urethral identification: a slightly longer echo time (TE) 3D-T2W SPACE sequence with a fat suppression technique (proposed sequence). Table 1 lists the acquisition parameters.

**Table 1:** Acquisition parameters

	3D-T2W (conventional)	Fat-suppressed 3D-T2W
Repetition time (ms)	1300	1500
Echo time (ms)	130	150
Flip angle (degree)	120 (constant)	150 (constant)
Field-of-view (mm)	300 × 300	250 × 250
Matrix (phase × read)	307 × 384	315 × 384
Acq. resolution (mm)	0.98 × 0.78 × 0.98	0.79 × 0.65 × 1.73
Rec. resolution (mm)	0.78 × 0.78 × 0.80	0.65 × 0.65 × 1.00
Bandwidth (Hz/pixel)	651	543
Echo spacing (ms)	4.00	4.28
Turbo factor	77	72
Number of slices	224	96
Slice oversampling (%)	14.3	33.3
Phase oversampling (%)	20	40
Number of averages	1.0	1.4
Excitation type	Non-selective	Slab-selective
Parallel imaging	GRAPPA of 4	GRAPPA of 3
Orientation	Coronal	Transversal
Fat-suppression	–	SPAIR
Acquisition time (m:sec)	5:28	5:45

*3D-T2W* three-dimensional T2-weighted, *GRAPPA* generalized autocalibrating partial parallel acquisition, *SPAIR* spectral attenuated inversion recovery

### Image analysis

Two medical physicists with 17 and 10 years of experience in radiation therapy independently conducted prostatic urethral contouring. According to our standard routine method in clinical practice, the prostatic urethra was contoured in a 4.0-mm-diameter region of interest (ROI). The contour analyses were conducted using MIM Maestro (MIM software ver. 6.9.4, EURO MEDITECH CO., LTD.). The Dice similarity coefficients (DSC) was calculated to compare interoperator variability for urethral contouring as an objective evaluation. As a subjective evaluation, both operators individually scored the prostatic urethral visibility in both images on a four-point scale (1 = non-identifiable; 2 = obscured, some effect on contouring; 3 = acceptable, no effect on contouring; 4 = clearly identifiable). In cases of inter-observer disagreement, final decisions were reached by consensus.

Additionally, the contrast-to-noise ratios (CNR) of both sequences were calculated and compared to quantitatively evaluate the image quality. First, both sequences were reconstructed into 2 mm slice-thickness sagittal planes, and a slice that could visualize the prostatic urethra was determined. The ROIs were carefully placed at the superior, middle, and inferior parts of the prostatic urethra on the proposed sequence and copied onto the conventional sequence. Another ROI was defined in a homogeneous location within the prostate gland (e.g., not including hyperplasia). The size of ROIs was 1.68–4.47 mm^2^ in the prostatic urethra and 0.14–0.21 cm^2^ in the prostate gland. The signal intensity (SI) and standard deviation (SD) were measured, and the CNR was calculated as follows:

1 $$\begin{array}{c}CNR=\frac{\left(Urethra SI-Prostate gland SI\right)}{Urethra SD}.\end{array}$$

### Statistical analysis

We used the Wilcoxon signed-rank test to compare the results for urethral identification (DSC, visibility score, and CNR) in both sequences. Spearman correlation coefficient (*ρ*) was calculated to assess the relationship between visibility score and CNR. A *p* < 0.05 was considered statistically significant. All statistical analyses were performed using the SPSS software (SPSS for Windows, version 28, IBM).

## Results

To demonstrate urethral identification enhancement using the proposed sequence, we conducted quantitative and qualitative comparisons with the conventional sequence. Table 2 summarizes each DSC value and visibility score in both sequences. The DSC values for interoperator variability of urethral contouring were 0.75 ± 0.07 and 0.81 ± 0.06 for conventional and proposed sequences, respectively (*p* = 0.001). The proposed sequence demonstrated significantly higher interoperator agreement than the conventional sequence. In the visual assessment, the visibilty scores were 2.6 ± 0.5 and 3.8 ± 0.6 for the conventional and proposed sequences, respectively (*p* = 0.001).

**Table 2:** Dice similarity coefficients and visibility scores in all patients

Case	Dice similarity coefficients		Visibility scores (by consensus)	
	3D-T2W	FS-3D-T2W	3D-T2W	FS-3D-T2W
1	0.82	0.89	3	4
2	0.64	0.81	2	4
3	0.79	0.81	3	4
4	0.76	0.87	3	4
5	0.78	0.78	2	3
6	0.72	0.84	2	4
7	0.70	0.79	2	4
8	0.82	0.84	3	4
9	0.79	0.83	3	4
10	0.59	0.64	2	2
11	0.80	0.80	3	4
12	0.75	0.82	3	4
13	0.79	0.81	3	4
Mean	0.75	0.81	2.6	3.8
SD	0.07	0.06	0.5	0.6
*p* value	*p* = 0.001		*p* = 0.001	

*p* < 0.05 was considered statistically significant

**Fig. 1:**
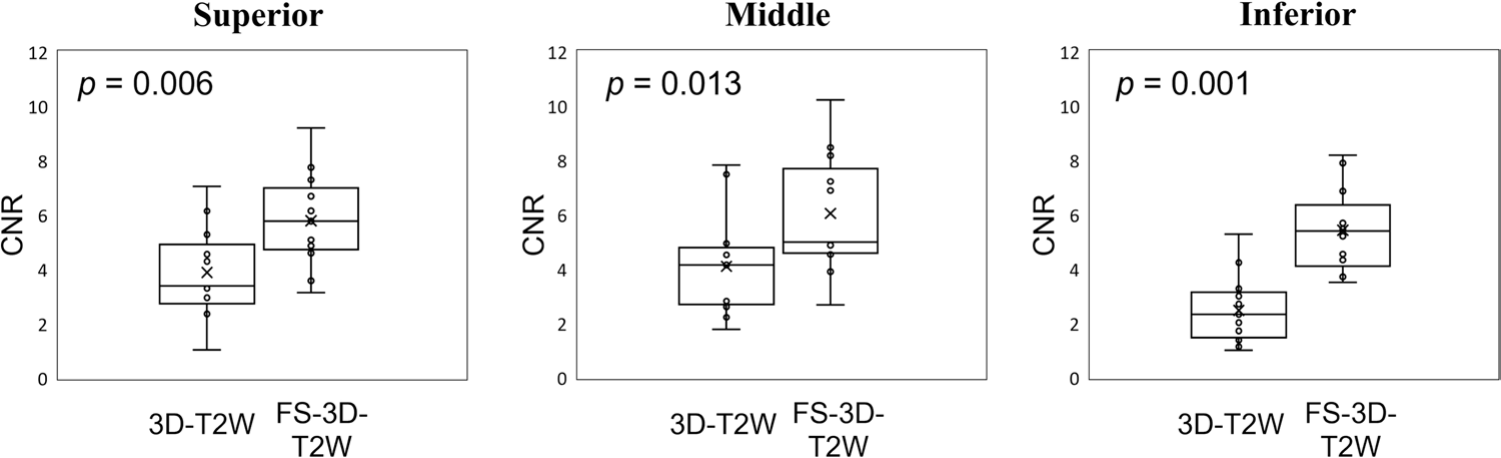
Box plots comparing the contrast-to-noise ratios (CNR) between conventional 3D T2-weighted (3D-T2W) and fat-suppressed (FS) 3D-T2W images for urethral identification. The CNRs of 3D-T2W vs. FS-3D-T2W images were 3.9 ± 1.6 vs. 5.8 ± 1.6 (*p* = 0.006), 4.1 ± 1.8 vs. 6.0 ± 2.1 (*p* = 0.013), and 2.5 ± 1.2 vs. 5.4 ± 1.4 (*p* = 0.001) in the superior, middle, and inferior parts of the prostatic urethra, respectively

In addition, to provide quantitative image evaluation, we calculated the CNR between the prostatic urethra and prostate gland. Figure 1 shows a box plot comparing both sequences. The CNR of conventional vs. proposed sequences were 3.9 ± 1.6 vs. 5.8 ± 1.6 (*p* = 0.006), 4.1 ± 1.8 vs. 6.0 ± 2.1 (*p* = 0.013), and 2.5 ± 1.2 vs. 5.4 ± 1.4 (*p* = 0.001) in the superior, middle, and inferior parts of the prostatic urethra, respectively. The CNRs obtained from the proposed sequence were significantly higher than those obtained from the conventional sequence. The conventional sequence showed no correlation (*ρ* = -0.085, *p* = 0.784), whereas the proposed sequence showed a positive correlation (*ρ* = 0.524, *p* = 0.066), although both had no significant difference.

**Fig. 2:**
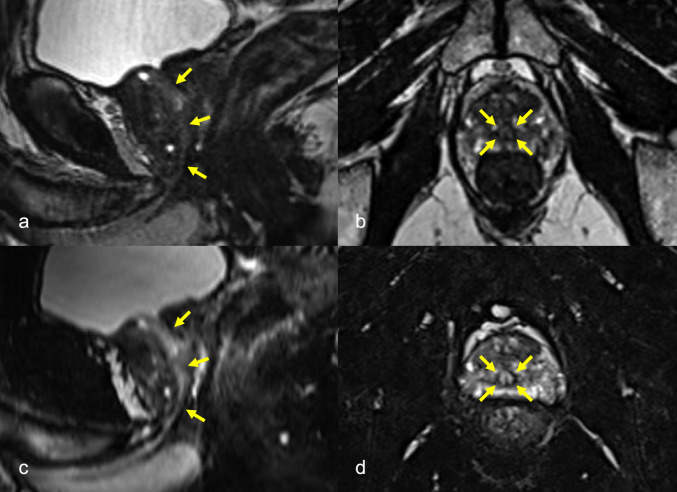
Images of a 76-year-old male in Case 1. The prostatic urethra is identified as a high-intensity tract (arrows) in the conventional 3D T2-weighted sagittal (**a**) and transverse (**b**) images, and it is more clearly identified in the proposed fat-suppressed 3D T2-weighted sagittal (**c**) and transverse (**d**) images

**Fig. 3:**
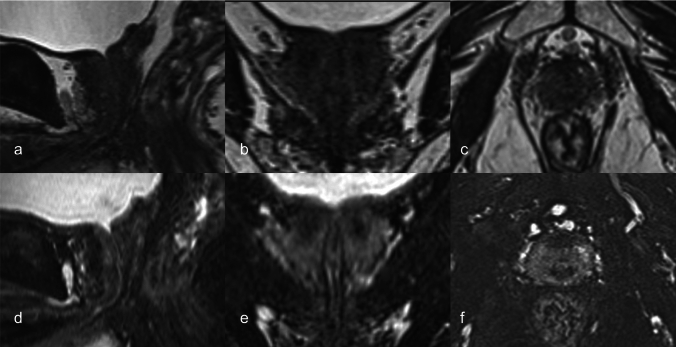
Images of a 68-year-old male in Case 7. The DSC value of the conventional 3D T2-weighted image was 0.70, and the visibility score was 2 (**a**–**c**). The urethra is a little difficult to visually identify. In contrast, the DSC value of the proposed fat-suppressed 3D T2-weighted image was 0.79, and the visibility score was 4 (**d**–**f**). The curve-reconstructed coronal image (**e**) shows a quite distinct boundary urethra/prostate

Figures 2 and 3 show the representative images. The prostatic urethra was identified as a high-intensity tract in both images. Figure 2 shows images of Case 1. Although the prostatic urethra is well identified in conventional sequence, it is more clearly identified in the proposed sequence. Figure 3 shows images of Case 7. The DSC value for the conventional sequence was 0.70 and the visibility score was 2. The urethra was difficult to identify visually. In contrast, the DSC value of the proposed sequence was 0.79 and the visibility score was 4. The curved reconstructed coronal image shows a distinct boundary between the urethra/prostate.

## Discussion

To demonstrate the improvement in urethral identification using the newly proposed fat-suppressed 3D-T2W sequence, we conducted quantitative and qualitative comparisons with a conventional sequence. Our proposed sequence showed superior urethral identification and provided high-CNR images, enabling accurate urethral contouring during radiotherapy planning.

**Fig. 4:**
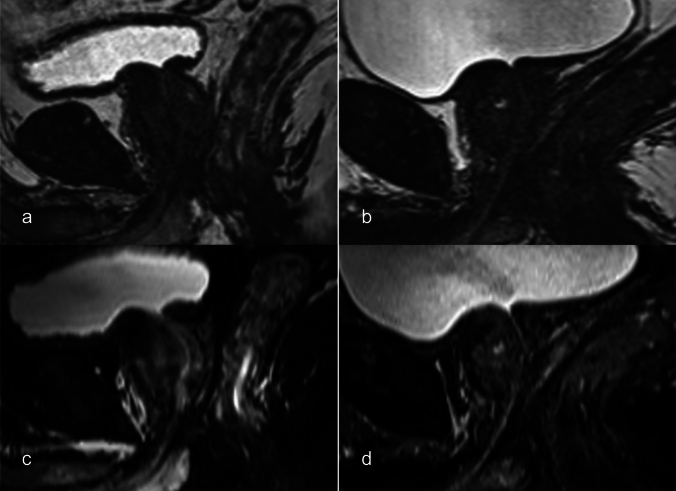
Sagittal images of a 76-year-old male in Case 2 (**a**, **c**). The DSC value of the conventional 3D T2-weighted image was 0.64 and the visibility score was 2, whereas the DSC value of the proposed fat-suppressed 3D T2-weighted image was 0.81, and the visibility score was 4. Sagittal images of a 67-year-old male in Case 4 (**b**, **d**). The DSC value of the conventional 3D T2-weighted image was 0.76, and the visibility score was 3, whereas the DSC value of the proposed fat-suppressed 3D T2-weighted image was 0.87, and the visibility score was 4

**Fig. 5:**
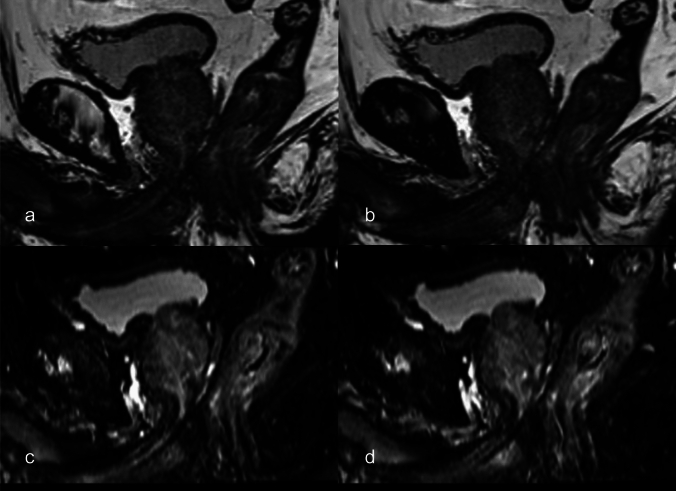
Images of a 75-year-old male in Case 10. The DSC value was 0.59 in the conventional 3D T2-weighted image (**a**, **b**) and 0.64 in the proposed fat-suppressed 3D T2-weighted image (**c**, **d**). The visibility score was 2 in both. This was the only case in which the prostatic urethra was obscured; this patient had benign prostatic hyperplasia, which may have been the cause

The DSC value of the proposed sequence is significantly higher than that of the conventional sequence. A DSC value of > 0.7 has been reported as demonstrating’good’ spatial and volumetric similarity [20]. Although the DSC of conventional sequence was comparable to that in the previous study [17] (0.75 in this study and 0.74 in the previous study), some cases were less than 0.7 in this study. Two cases with marked differences between the two sequences are shown in Fig. 4. In Case 2, for example, the DSC value was 0.64 in the conventional sequence, whereas 0.81 in the proposed sequence, the visibility score was 4, indicating a clear identification (Fig. 4a, c). In most other cases, the proposed sequence scored > 0.8, which is a higher agreement rate than that of the conventional sequence. However, the value was not comparable to that of CT with a catheter (0.93 [17]) but was equivalent to that of CT urethrogram (0.81 [21]). In the visual assessment, the visibility score of the proposed sequence yielded mostly 4, although it did not yield 4 in a previous study [17]. The use of this approach will provide more confidence in urethral contouring. The worst DSC value (Case 10) had a visibility score of 2 and difficulty in identifying the prostatic urethra (Fig. 5). The patient had benign prostatic hyperplasia, which might have been the cause. A previous study have reported that the prostatic urethra may be compressed and displaced due to large benign prostatic hyperplastic nodules [12]. In addition, high prostate signal due to inflammation may obscure urethral visualization, which may be a less-effective scenario for the proposed sequence.

The CNR of the proposed sequence was significantly better than that of the conventional sequence, resulting in a good visual assessment. The conventional sequence had no correlation between visibility score and CNR, suggesting less reliability for urethral identification. In contrast, the proposed sequence showed a positive correlation, although not significant, which may indicate higher reliability for urethral identification. This technique applies fat suppression to the standard 3D-T2W sequence to enhance urethral visualization. It is a simple method that does not require specialized sequence design, making it readily applicable to other facilities. Many previous studies have described the need for further improvements in urethral identification [15, 17, 21], and our proposed approach may be a solution.

In the proposed sequence, fat suppression technique altered the dynamic range of the image and highlighted the urethral water signal. In addition, the proposed sequence employs a slightly longer TE than the conventional sequence. Applying a long TE may help enhance the water signal (i.e., heavily-T2W sequences), which is widely used in MR hydrography for clinical applications [22, 23]. These approaches could have contributed to the improvement in visibility scores and CNR.

This study has some limitations. First, the sample size is small. Second, we did not investigate the detailed imaging parameters. Although a longer TE may improve urethral visualization, if it is also used for entire prostate contouring, an excessively long TE may not be appropriate because of the reduced signal from the surrounding tissues. Therefore, we adapted a slightly longer TE (150 ms) than standard T2W sequence in this study. The optimization of the imaging parameters is a future challenge.

## Conclusion

This study has demonstrated that the newly proposed fat-suppressed 3D-T2W sequence provides superior DSC values, visibility scores, and high-CNR images in prostatic urethra identification. Our findings suggest that this approach could be a valuable noninvasive method for enhancing prostatic urethral identification in radiotherapy planning.

## Data Availability

The data that support the findings of this study are not openly available due to reasons of sensitivity and are available from the corresponding author upon reasonable request.
